# The association between parameters of physical activity and olfactory function—the importance of frequency and moderate intensity

**DOI:** 10.3389/fspor.2024.1394858

**Published:** 2024-06-05

**Authors:** Khoosheh Namiranian, Alexandre-Charles Gauthier, Jo-Anne Gilbert, Marie-Eve Mathieu

**Affiliations:** ^1^ School of Kinesiology and Physical Activity Sciences, Faculty of Medicine, Université de Montréal, Montréal, QC, Canada; ^2^ Azrieli Research Center—Sainte-Justine University Hospital Center, Chemin de la Côte-Sainte-Catherine, Montréal, QC, Canada

**Keywords:** duration, frequency, intensity, olfaction, physical activity, smell, volume

## Abstract

**Introduction:**

Overall physical activity (PA) has been linked to decreased olfactory dysfunction and could improve olfactory function. Additionally, decreased olfactory function can contribute to reduced overall PA levels, given its association with neurodegenerative disorders. This study aims to examine the relationship between specific PA parameters (duration, frequency, intensity) and olfactory function in adults.

**Methods:**

A total of 3,527 participants from the National Health and Nutrition Examination Survey (NHANES) 2013–2014 underwent assessments for weekly PA duration, frequency, and intensity, alongside a smell test (including odors such as chocolate, strawberry, grape, onion, smoke, natural gas, leather, and soap). Correlation analyses and binary logistic regressions using SPSS were used to evaluate associations.

**Results:**

The total smell score exhibited small yet significant positive correlations with the duration, frequency, and volume of moderate PA (correlation coefficients ranging between 0.05 and 0.08; all *p* ≤ 0.05) and frequency of vigorous PA (correlation coefficient of 0.05; *p* < 0.05). For moderate PA, the duration, frequency, and volume were significantly and positively associated with the ability to correctly detect the smell of grapes while the frequency was significantly and positively associated with the ability to identify smoke and leather odors (odds ratios ranging from 1.01 to 1.07; *p* < 0.05). For vigorous PA, the frequency of PA was positively associated with the detection of grape smell (odds ratio of 1.05; *p* < 0.05).

**Conclusion:**

Some parameters of an active lifestyle are associated with improved odds of accurately identifying odors by up to 7.4%. Moderate PA duration, frequency, and volume were linked to better olfactory scores, while high-intensity PA had limited associations.

## 1 Introduction

An intact olfactory system is crucial for maintaining a good quality of life (1). In addition to detecting pleasant and unpleasant odors, our sense of smell plays an essential role in protecting us against environmental hazards, such as gas leaks, and regulating eating behaviors (2). For instance, the inability to smell food can impair taste perception, which can negatively affect appetite and lead to malnutrition (3). Moreover, olfactory loss or dysfunction could negatively impact physical and mental well-being, safety, appetite, and nutritional status (4, 5). Olfactory impairments are highly prevalent in the general population, as various factors can affect olfactory function, such as hunger, anthropometrics, sociodemographics, and health characteristics (5, 6). For example, higher levels of hunger can increase sensitivity to certain odors (7, 8), while a high body mass index has been associated with decreased olfactory sensitivity (9). Olfactory function can also decrease with age (10, 11) and is typically lower in males (12) and patients with neurological and cognitive disorders (13). These impairments became particularly important during the recent COVID-19 pandemic with an average of 47% of patients reporting chemosensory dysfunction (15).

In humans, regular PA in older adults has been associated with a lower incidence of olfactory dysfunction that was volume-dependent. In our study, the participants who engaged in more PA have a lower risk of olfactory dysfunction over 10 years (16). In 2016, Hoffman et al. (12) observed that a minimal amount of moderate-to-vigorous PA, with a threshold of 10 continuous minutes of moderate-to-vigorous activity for ≥3 days/week, was associated with a lower prevalence of olfactory dysfunction (8.9% for active individuals vs. 20% for inactive individuals). Other researchers have observed that regular participation in activities such as Tai Chi, dancing, or running (for over a year, >3 times/week, with each session lasting >30 min) can significantly improve odor detection and identification compared to individuals who rely solely on walking or engage in no PA (17). Furthermore, it has been observed that a higher frequency of these activities (i.e., ≥3 times/week or more, compared to one to two times/week or not at all) is associated with a greater reduction in olfactory dysfunction (17). In individuals with asthma and chronic sinusitis, olfactory function, particularly gas odor detection, was improved following a 12-week aerobic exercise program combined with nasal breathing exercises (18).

Despite the recent studies supporting the fact that PA can help maintain or improve olfaction, it is possible that in some cases reduced olfaction may contribute to, or be a marker of, subsequent reduced PA. For example, alterations in olfactory function have been proposed as early biomarkers of neurodegenerative disorders, such as Parkinson's and Alzheimer's disease (19), while these conditions are associated with disabling factors such as motor symptoms, rigidity, and postural instability (19).

To the best of our knowledge, no study has comprehensively addressed specific PA parameters concerning olfactory function, as our group recently did for taste (20). In the latter study, we found that frequency and duration of VPA were generally more important than moderate-intensity parameters and that the associations differed depending on the taste being tested. The subgroup analysis also showed that individuals who were not living with advanced stages of obesity had better associations between an active lifestyle and taste integrity. Knowledge of the relationship between PA parameters and chemosensory (taste and smell) integrity is important to support intervention strategies and/or to understand specific changes following olfactory impairment. Therefore, the present study aims to investigate the association between distinctive PA parameters and olfactory function, taking advantage of the National Health and Nutritional Examination Study (NHANES) study that provides information on PA's intensity, duration, frequency, and volume. A secondary objective of the study was to study associations according to body weight status.

## 2 Materials and methods

### 2.1 Study design and population

The NHANES aims to assess the health and nutrition status of a representative sample of the US population. In this study, a secondary analysis of NHANES data from the 2013–2014 cycle that includes smell assessments was conducted. A total of 3,527 adults aged between 40 and 80 years who completed the smell test and answered the self-report questionnaire on PA were included. Body measurements [body weight in kilograms and standing height in meters were used to calculate body mass index (kg/m^2^)] were collected by the investigator team. The body mass index of the participants was used as the main stratifying variable and was divided into four different categories: normal weight (<24.99 kg/m^2^), overweight (25.00–29.99 kg/m2), obesity class 1 (30.00–34.99 kg/m^2^), and obesity classes 2 and 3 (>35.00 kg/m^2^). The NHANES agreement received approval from the National Center for Health Statistics Research Ethics Committee, and all adult participants provided written informed consent. Each participant was assessed using a computer-assisted personal interview system operated by registered health technicians.

### 2.2 Smell test

This study used the eight-item NHANES pocket test (PST, Sensonics International, Inc., Haddon Heights, NJ, USA) to assess olfactory function. This test incorporates four nutrient-related odors (chocolate, strawberry, grape, and onion), two warning odors (smoke and natural gas), and two common household odors (leather and soap). The test is fully described in Hoffman's 2016 paper (12). Based on the number of correct identifications of odors, the total smell score ranged from 0 to 8. A score of 0–5 (≥3 incorrect responses) indicates an olfactory disorder, and a score of 6–8 is considered normal.

### 2.3 PA questionnaire

Within the NHANES, the participants completed a self-report questionnaire on PA based on the Global PA Questionnaire (21). This questionnaire covered daily, leisure, and sedentary activities and was administered at home before the physical examination. For the analysis, moderate-intensity activities were those that caused small increases in breathing or heart rate, such as brisk walking or carrying light loads for at least 10 min. Activities that caused an increase in heart rate or respiration rate—such as carrying or lifting heavy loads, digging, or building work—continuously for at least 10 min during the week were considered vigorous PA (21). PA duration was defined in bouts of 10 min for OR calculations, and the number of PA sessions determined the frequency of PA in a typical week (day/week). The volume of PA was calculated by metabolic equivalent (METs*h/week), with four METs attributed to moderate activity and eight METs for vigorous work-related activity (21). For the presentation, the total volume of PA in logistic regression was divided by 100.

### 2.4 Statistical analysis

Pearson correlations and binary logistic regressions were used to estimate the associations between olfaction and PA parameters. Age and sex were considered covariates in logistic regression because they are associated with both olfactory function and PA. Odds ratios (ORs) represented the constant effect of PA on the probability of detecting odors correctly in the eight-item NHANES pocket smell test to evaluate the effect of PA parameters on the chance of detecting odors correctly. A *p*-value of ≤0.05 was considered statistically significant. The statistical analyses were performed using SPSS 27.0 (SPSS Inc., USA).

## 3 Results

A total of 3,527 participants (47.6% men aged 40–80 years old) responded to the PA questionnaire and performed the smell test (Table 1). They were categorized as underweight/normal weight (26.5%), overweight (34.1%), obesity class 1 (21.7%), or obesity class 2 or 3 (16.6%).

**Table 1:** Characteristics of the participants.

	Mean ± SD	*n*
Age (years)	59.0 ± 12.0	3,527
Sex (male)		1,680
Body mass index (kg/m^2^)	29.38 ± 6.90	3,486
Total volume (METs*h/week)	27.09 ± 72.94	3,517
Moderate intensity		
Duration (min/week)	40.6 ± 96.1	3,517
Frequency (days/week)	1.20 ± 2.15	3,521
Volume (METs*h/week)	12.38 ± 33.35	3,517
Vigorous intensity		
Duration (min/week)	25.8 ± 83.4	3,522
Frequency (days/week)	0.60 ± 1.58	3,526
Volume (METs*h/week)	14.69 ± 53.97	3,522
Total smell	6.61 ± 1.42	3,519
Chocolate	0.82 ± 0.38	3,527
Strawberry	0.79 ± 0.40	3,525
Grape	0.62 ± 0.48	3,519
Onion	0.94 ± 0.23	3,519
Gas	0.86 ± 0.34	3,523
Smoke	0.86 ± 0.34	3,519
Leather	0.76 ± 0.42	3,520
Soap	0.92 ± 0.26	3,520

METs, metabolic equivalents; PA, physical activity; SD, standard deviation.

**Figure 1:**
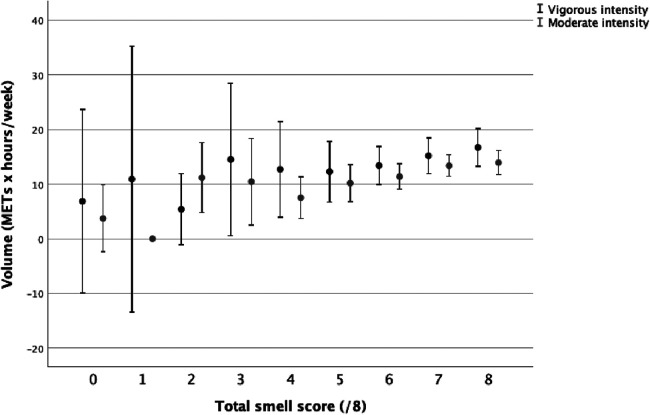
Total smell score by volume of moderate and vigorous physical activity.

**Figure 2:**
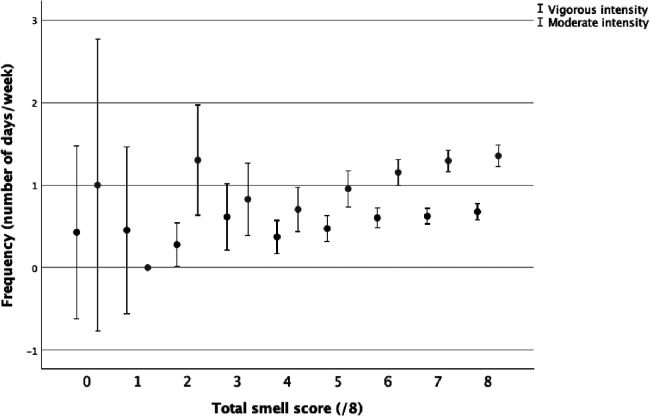
Total smell score by frequency of moderate and vigorous physical activity.

**Figure 3:**
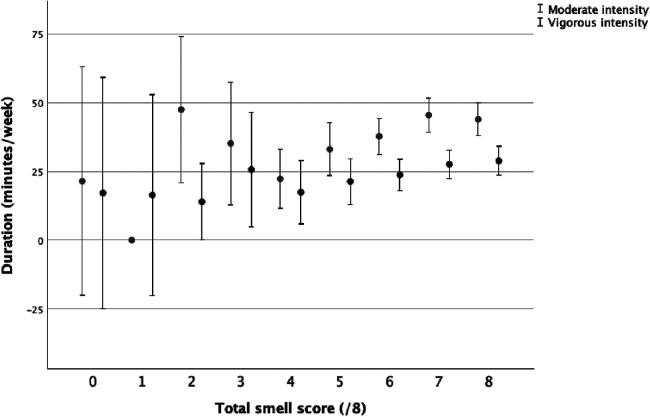
Total smell score by number of days of moderate and vigorous physical activity.

The total volume of PA was correlated to the total smell score (*r* = 0.047, *p* = 0.006). Small and positive correlations were present between the total smell score and duration, frequency, and volume of moderate PA (Table 2). The total smell score was positively correlated with the frequency of vigorous PA (Table 2). The total smell scores by volume of moderate and vigorous PA (Figure 1), frequency of moderate and vigorous PA (Figure 2), and duration of moderate and vigorous PA (Figure 3) are presented graphically.

**Table 2:** Correlations between total smell score and physical activity parameters.

Duration (min/week)			Frequency (days/week)			Volume (METs*h/week)		
*n*	*r*	*p*-value	*n*	*r*	*p*-value	*n*	*r*	*p*-value
Moderate physical activity								
3,509	0.050	**0.003**	3,513	0.076	**<0**.**001**	3,509	0.051	**0**.**002**
Vigorous physical activity								
3,514	0.032	0.060	3,518	0.049	**0**.**004**	3,514	0.032	0.060

*n*, number of participants; *r*, correlation coefficient. Bold represents significant *p*-value.

When the analyses were stratified by body weight status, there was no significant correlation for underweight/normal weight individuals. However, there were three significant correlations for individuals living with overweight [number of days of moderate (*r* = 0.097, *p* < 0.001) and vigorous (*r* = 0. 076, *p* = 0.009) intensity PA per week and duration of vigorous PA per week (0.060, *p* = 0.039)], one for individuals living with class 1 obesity [duration of vigorous PA per week (*r* = 0.072, *p* = 0.047)], and none for individuals living with obesity classes 2–3.

The results of the logistic regressions for PA total volume with individual odors are presented in Table 3. The results showed no significant association between all eight olfactory detections and the total volume of PA. The absence of significant association was confirmed for analysis performed per subgroups of bodyweight status.

**Table 3:** The effect of total physical activity volume on the ability to correctly detect the odors.

Odor	*n*	OR	95% CI	*p*-value
Chocolate	3,517	0.958	(0.850–1.079)	0.476
Strawberry	3,515	0.979	(0.887–1.120)	0.959
Grape	3,509	1.068	(0.966–1.181)	0.198
Onion	3,509	1.056	(0.830–1.345)	0.655
Gas	3,509	1.122	(0.943–1.334)	0.193
Smoke	3,513	1.066	(0.912–1.247)	0.421
Leather	3,510	1.108	(0.979–1.255)	0.105
Soap	3,510	0.953	(0.797–1.140)	0.596

OR is for raising 100 units of PA total volume, expressed in METs*h/week.

While total PA level yielded no significant results, specific PA parameters emerged. The duration of moderate PA (bouts of 10 min/day) showed a small and significant association with the ability to correctly detect the smell of grapes [OR = 1.010, CI 95% (1.002–1.017), *p* = 0.015] (Table 4). The frequency of moderate PA (day/week) showed small associations with the detection of grape smell [OR = 1.047, CI 95% (1.013–1.082), *p* = 0.007], smoke [OR = 1.074, CI 95% (1.019–1.131) *p* = 0.008], and leather [OR = 1.060, CI 95% (1.019–1.103), *p* = 0.004]. The volume of moderate PA (METs*h/week) was associated with the ability to correctly detect the smell of grapes [OR = 1.002, CI 95% (1.000–1.005), *p* = 0.028]. For vigorous PA, the frequency of PA (day/week) was associated with the detection of grape smell [OR = 1.051, CI 95% (1.004–1.101), *p* = 0.034].

**Table 4:** Relation between participants’ ability to correctly identify specific odors and moderate and vigorous physical activity parameters.

		Chocolate		Strawberry		Grape		Onion		Gas		Smoke		Leather		Soap	
		*n*	OR (95% CI)	*n*	OR (95% CI)	*n*	OR (95% CI)	*n*	OR (95% CI)	*n*	OR (95% CI)	*n*	OR (95% CI)	*n*	OR (95% CI)	*n*	OR (95% CI)
Moderate Physical activity	Duration (bouts 10 min/week)	3,517	0.999 (0.990–1.009)	3,515	0.997 (0.989–1.006)	3,509	**1.010** (**1.002–1.017**)	3,509	1.009 **(**0.989–1.028)	3,509	1.002 (0.991–1.014)	3,513	1.004 (0.993–1.016)	3,510	1.008 (0.993**–**1.016)	3,510	1.003 (0.999–1.018)
	Frequency (days/week)	3,521	1.014 (0.972–1.057)	3,519	0.992 (0.954–1.032)	3,513	**1.047** (**1.013–1.082**)	3,513	1.057 (0.976–1.145)	3,513	1.001 (0.954–1.051)	3,517	**1.074** (**1.019–1.131**)	3,514	**1.060** (**1.019–1.103**)	3,514	1.031 (0.967–1.099)
	Volume (METs*h/week)	3,517	1.000 (0.997–1.002)	3,515	0.999 (0.997–1.002)	3,509	**1.002** (**1.000–1.005**)	3,509	1.004 (0.997–1.010)	3,509	1.000 (0.997–1.004)	3,513	1.002 (0.999–1.006)	3,510	1.002 (0.999–1.005)	3,510	1.000 (0.996–1.004)
Vigorous physical activity	Duration (bouts 10 min/week)	3,522	0.999 (0.997–1.001)	3,520	1.000 (0.998–1.002)	3,514	1.000 (0.999–1.002)	3,514	1.000 (0.996–1.004)	3,514	1.003 (0.999–1.006)	3,518	1.000 (0.998–1.003)	3,515	1.001 (0.999–1.004)	3,515	0.999 (0.996–1.002)
	Frequency (days/week)	3,526	0.985 (0.932–1.042)	3,524	0.984 (0.934–1.038)	3,518	**1.051** (**1.004–1.101**)	3,518	1.027 (0.922–1.142)	3,518	1.052 (0.976–1.134)	3,522	1.059 (0.985–1.139)	3,519	1.041 (985–1.099)	3,519	0.962 (0.887–1.045)
	Volume (METs*h/week)	3,522	0.999 (0.998–1.001)	3,520	1.000 (0.999–1.002)	3,514	1.000 (0.999–1.002)	3,514	1.000 (0.997–1.003)	3,514	1.002 (1.000–1.005)	3,518	1.000 (0.999–1.003)	3,515	1.000 (0.998–1.003)	3,515	1.000 (0.998–1.002)

OR, odds ratio; CI, confidence interval. Bold highlights significant *p*-value (*p* < 0.05).

Specificities were found when the analysis was stratified by a subgroup of body weight status. For underweight/normal weight individuals, significant ORs for chocolate of 0.998 (*p* = 0.010) for duration and of 0.994 (0.022) for volume of MPA were obtained, while they were of 1.003 (*p* = 0.006) for duration and 1.008 (*p* = 0,012) for volume of MPA for grape. For overweight individuals, the OR for frequency of MPA was 1.097 (*p* = 0.037) for smoke. For individuals belonging to obesity class 1, the ORs for frequency of MPA were 1.355 (*p* = 0.020) for onion, 1.121 (*p* = 0.016) for leather, and 1.221 (*p* = 0.023) for soap. No significant ORs were found for VPA or in individuals belonging to obesity classes 2–3.

## 4 Discussion

This study aimed to investigate the association between frequency, duration, and volume of PA performed at both moderate and vigorous intensities and the detection of eight distinct odors related to nutrition, warning, and everyday household groups. Our findings first indicated that the total volume of PA was rarely associated with smell outcomes, and all three components (duration, frequency, and volume) of moderate intensity are worth considering. Among those, frequency was more often associated with better smell recognition. High PA intensity components also deserve attention but generally yield borderline significant results, with lower associations or fewer significant ORs than those found for moderate PA components. Correlation and OR analysis also showed that people who were overweight or who belonged to obesity class 1 had higher associations between their PA levels and olfactory performances.

In the study, the ORs for duration indicated the increased probability of success for every increment of a 10-min block of PA per week, while the ORs for frequency represented the increased probability of success for each additional day of PA practice per week. The ORs for volume reflected the increased probability of success for every additional MET per week related to PA practice. In this case, every additional block of moderate PA was associated with a 1% increase in the odds of accurately identifying the smell of grape, while frequency and volume were associated with increases of 4.7% and 0.2%, respectively. The frequency of moderate PA was also linked to increases of 7.4% and 6% in the odds of accurately identifying the odors of smoke and leather, respectively. Moreover, few advantages emerged from the practice of high-intensity PA, where the only observed increase in the probability of successfully identifying the smell of grape (+5.1%) was seen with its frequency. According to this data set, each component of the active lifestyle investigated could increase the odds of accurately identifying odors by up to 7.4%.

As mentioned previously, Hoffman et al. (12) had previously observed that individuals who regularly engaged in PA had at least half the prevalence of smell impairment compared to inactive individuals. Regular exercise, defined as at least 10 continuous minutes of vigorous- or moderate-intensity activity for ≥3 days/week, seemed to confer protection against smell impairment (12). Another study, which examined the olfactory function over 10 years, also mentioned that regular exercise might positively affect olfactory function in older adults (16). The present study observed similar results with various components of PA. While Sollai and colleagues (20) had previously reported a positive correlation between olfactory scores and the number of hours devoted to PA per week, we observed a significant association with the chance of detecting odors correctly but more often for moderate intensity. This finding remained consistent for both total smell scores and specific odors (i.e., grape). It is worth noting that the importance of moderate intensity appears specific to smell integrity. In contrast, a secondary analysis conducted by our group, using the same dataset, revealed that more intense PA is of greater interest for taste integrity (20), which is the other sense included in chemosensory response. The results of this study align with those of the longitudinal studies assessing this matter (12, 14), and our secondary analysis on taste (20) also revealed that the frequency of PA is essential to consider for chemosensory integrity. The present study highlights frequency as the most important parameter, both for moderate and vigorous PA, to obtain better total scores and results specific to odors. For all three types of odors (warning, food, and household), the better profile occurred when frequent PA was performed at a moderate level. The importance of frequency may explain why vigorous PA did not yield as interesting results as moderate PA because it is performed 50% less frequently, as shown in Table 1. Given that the amounts of PA were similar for moderate and vigorous PA, we can hypothesize that for a given amount of PA, lower-intensity PA performed more frequently may be more interesting for olfactory integrity than higher-intensity PA performed less frequently.

The differential response by a subgroup of body weight status suggests that an active lifestyle is not associated with different olfactory outcomes or that it produces mixed results for individuals without excess body weight (i.e., underweight and normal weight), some positive results for individuals living with overweight, and more substantial positive findings for individuals living with obesity class 1. In contrast to recent work from our group on taste and PA, where adverse associations were found for individuals who belonged to obesity classes 2 and 3 (20), it was interesting that no adverse effects were found for these individuals for smell. While the relationship between obesity and olfactory integrity is increasingly being investigated, it remains unclear why the associations between PA and olfaction differ according to body mass index groups. The fact that higher associations were found between PA and olfaction in individuals with obesity, at least in class 1, is intriguing, and further studies are needed in this area of investigation.

Doty (23), in 2019, stated that the evidence supporting strategies such as exercise to combat age-related dysfunction in smell was compelling. However, it remains unclear if chronic practice of PA could reverse deficits in chemosensation once they are present. The current study adds valuable information regarding which intensity of PA could be beneficial. In this context, moderate PA was associated with better odor recognition than high-intensity PA. In NHANES 2013–2014, it was reported that the prevalence of smell disorder was associated with overall PA volume (METs*min/week), with more impairments in lower tertiles of PA (24). In our study, vigorous PA volume was not significantly associated with odor recognition, while moderate PA volume was associated with correctly detecting food-related odors. It seems prudent at this stage to conduct randomized controlled trials where the intensity of PA is strictly controlled to better understand the role that PA can play in both olfactory preservation and improvement.

A growing body of literature using longitudinal and experimental designs supports the fact that PA may be a strategy to maintain or improve smell, with potential mechanisms including reduced hypertension, reduced cell and neurotransmitter loss, and hormonal modulation including insulin sensitivity (25–29). However, the cross-sectional design of the current study does not allow us to confirm the direction of the association. As mentioned in the introduction, it is theoretically possible that smell reduction may contribute to, or be a marker of, subsequent reduced PA levels. In addition, a recent systematic review conducted by our group highlighted that odor exposure in most cases acutely improves PA performance (30). If performance is improved, this could theoretically help to maintain PA levels, and therefore the reduction in smell could reduce PA levels. At this stage, these remain hypotheses to be tested.

### 4.1 Strengths and limitations

The current study utilized a large sample size and objectively assessed smell using the eight-item NHANES pocket test, which includes various odors categorized as nutrition, warning, and common household odors. Additionally, the PA questionnaire allows for a distinctive analysis of PA parameters. Furthermore, covariates affecting PA and olfactory function (i.e., sex and age) were considered while analyzing the data. Although our study assessed the smell of a wide range of adults aged between 40 and 80 years, future investigations may benefit from including younger and older individuals to cover the full spectrum. Older adults may have pathological changes in the central olfactory processing area due to age or cognitive disorders related to olfactory function (6), which could affect odor smell scales. Another limitation of the current study is that there may be a sampling bias, as participation was voluntary, and that very few odors were used with a very narrow cutoff, probably to compensate for the time required to test such a large sample size. As this is the first study to explore the possible association between specific PA parameters and smell detection, simple analyses have been conducted, and results could underline false positive results.

## 5 Conclusion

Based on secondary analyses of NHANES data, associations between total smell score and PA total volume and parameters of mainly moderate intensity were observed. Specifically, at moderate intensity, the frequency had more associations with accurate smell detection in all three odor categories (nutrition, warning, and common household), while the frequency of vigorous PA was associated only with better results for the nutrition group odors. Given that moderate levels of PA have been found to enhance the ability to detect odors correctly, it could be advisable to consider moderate PA for interventions and future research.

## Data Availability

Publicly available datasets were analyzed in this study. This data can be found here: https://wwwn.cdc.gov/nchs/nhanes/continuousnhanes/default.aspx?BeginYear=2013.

## References

[1] Auffarth B. Understanding smell—the olfactory stimulus problem. Neurosci Biobehav Rev. (2013) 37(8):1667–79. 10.1016/j.neubiorev.2013.06.009 23806440

[2] Stevenson RJ. An initial evaluation of the functions of human olfaction. Chem Senses. (2010) 35(1):3–20. 10.1093/chemse/bjp083 19942579

[3] Boesveldt S de Graaf K. The differential role of smell and taste for eating behavior. Perception. (2017) 46(3-4):307–19. 10.1177/0301006616685576 28056650

[4] Doty RL Kamath V. The influences of age on olfaction: a review. Front Psychol. (2014) 5(20):72845. 10.3389/fpsyg.2014.00020 PMC3916729 24570664

[5] Doty RL. Systemic diseases and disorders. Handb Clin Neurol. (2019) 164:361–87. 10.1016/B978-0-444-63855-7.00021-6 31604558

[6] DeVere R. Disorders of taste and smell. Continuum (Minneap Minn). (2017) 23(2):421–46. 10.1212/CON.0000000000000463 28375912

[7] Rolls ET. Taste and smell processing in the brain. Handb Clin Neurol. (2019) 164:97–118. 10.1016/B978-0-444-63855-7.00007-1 31604566

[8] Stafford LD Welbeck K. High hunger state increases olfactory sensitivity to neutral but not food odors. Chem Senses. (2011) 36(2):189–98. 10.1093/chemse/bjq114 20978137

[9] Skrandies W Zschieschang R. Olfactory and gustatory functions and its relation to body weight. Physiol Behav. (2015) 142:1–4. 10.1016/j.physbeh.2015.01.024 25619950

[10] Schubert CR Cruickshanks KJ Klein BE Klein R Nondahl DM. Olfactory impairment in older adults: five-year incidence and risk factors. Laryngoscope. (2011) 121(4):873–8. 10.1002/lary.21416 21298645 PMC3063862

[11] Kondo K Kikuta S Ueha R Suzukawa K Yamasoba T. Age-related olfactory dysfunction: epidemiology, pathophysiology, and clinical management. Front Aging Neurosci. (2020) 12:208. 10.3389/fnagi.2020.00208 32733233 PMC7358644

[12] Hoffman HJ Rawal S Li CM Duffy VB. New chemosensory component in the US National Health and Nutrition Examination Survey (NHANES): first-year results for measured olfactory dysfunction. Rev Endocr Metab Disord. (2016) 17:221–40. 10.1007/s11154-016-9364-1 27287364 PMC5033684

[13] Tian Q An Y Kitner-Triolo MH Davatzikos C Studenski SA Ferrucci L Associations of olfaction with longitudinal trajectories of brain volumes and neuropsychological function in older adults. Neurology. (2023) 100(9):e964–74. 10.1212/WNL.0000000000201646 36460474 PMC9990434

[14] Schubert CR Fischer ME Pinto AA Klein BE Klein R Cruickshanks KJ. Odor detection thresholds in a population of older adults. Laryngoscope. (2017) 127(6):1257–62. 10.1002/lary.26457 28000220 PMC5444983

[15] Borsetto D Hopkins C Philips V Obholzer R Tirelli G Polesel J Self-reported alteration of sense of smell or taste in patients with COVID-19: a systematic review and meta-analysis on 3563 patients. Rhinology. (2020) 58(5):430–6. 32626853 10.4193/Rhin20.185

[16] Schubert CR Cruickshanks KJ Nondahl DM Klein BE Klein R Fischer ME. Association of exercise with lower long-term risk of olfactory impairment in older adults. JAMA Otolaryngol Head Neck Surg. (2013) 139(10):1061–6. 10.1001/jamaoto.2013.4759 24135745 PMC3855446

[17] Zhang C Li D Wang X. Role of physical exercise type in olfactory deterioration in ageing. Rhinology. (2020) 58(2):145–50. 10.4193/Rhin19.274 32249853

[18] Zarneshan A. Effects of regular aerobic with nasal breathing exercise training on olfactory rehabilitation in asthmatic patients with chronic rhino sinusitis. J Rehabil Sci Res. (2020) 7(4):178–83.

[19] Dan X Wechter N Gray S Mohanty JG Croteau DL Bohr VA. Olfactory dysfunction in aging and neurodegenerative diseases. Ageing Res Rev. (2021) 70:101416. 10.1016/j.arr.2021.101416 34325072 PMC8373788

[20] Gauthier AC Dupont F Mathieu ME. Association between physical activity and taste–the advantage of increased intensity for some but not all individuals. PLoS One. (2023) 18(12):e0295173. 10.1371/journal.pone.0295173 38150407 PMC10752529

[21] Centers for Disease Control and Prevention (CDC). National Center for Health Statistics (NCHS). National Health and Nutrition Examination Survey Questionnaire Instruments. Hyattsville, MD: U.S. Department of Health and Human Services, Centers for Disease Control and Prevention, [2013-2014] (2006). Available online at: https://wwwn.cdc.gov/nchs/nhanes/continuousnhanes/questionnaires.aspx?BeginYear=2013 (accessed November 15, 2023).

[22] Sollai G Crnjar R. Age-related olfactory decline is associated with levels of exercise and non-exercise physical activities. Front Aging Neurosci. (2021) 13:695115. 10.3389/fnagi.2021.695115 34504418 PMC8423134

[23] Doty RL. Treatments for smell and taste disorders: a critical review. Handb Clin Neurol. (2019) 164:455–79. 10.1016/B978-0-444-63855-7.00025-3 31604562

[24] Liu G Zong G Doty RL Sun Q. Prevalence and risk factors of taste and smell impairment in a nationwide representative sample of the US population: a cross-sectional study. BMJ Open. (2016) 6(11):e013246. 28157672 10.1136/bmjopen-2016-013246 PMC5129069

[25] Catamo E Tornese G Concas MP Gasparini P Robino A. Differences in taste and smell perception between type 2 diabetes mellitus patients and healthy controls. Nutr Metab Cardiovasc Dis. (2021) 31(1):193–200. 10.1016/j.numecd.2020.08.025 33500104

[26] Schiffman SS. Taste and smell losses in normal aging and disease. JAMA. (1997) 278(16):1357–62. 10.1001/jama.1997.03550160077042 9343468

[27] Erickson KI Voss MW Prakash RS Basak C Szabo A Chaddock L Exercise training increases size of hippocampus and improves memory. Proc Natl Acad Sci U S A. (2011) 108(7):3017–22. 10.1073/pnas.1015950108 21282661 PMC3041121

[28] Palouzier-Paulignan B Lacroix MC Aimé P Baly C Caillol M Congar P Olfaction under metabolic influences. Chem Senses. (2012) 37(9):769–97. 10.1093/chemse/bjs059 22832483 PMC3529618

[29] Poessel M Freiherr J Wiencke K Villringer A Horstmann A. Insulin resistance is associated with reduced food odor sensitivity across a wide range of body weights. Nutrients. (2020) 12(8):2201. 10.3390/nu12082201 32721994 PMC7468861

[30] Cournoyer M Maldera A Gauthier AC Dal Maso F Mathieu ME. Effect of odor stimulations on physical activity: a systematic review. Physiol Behav. (2023) 114408. 10.1016/j.physbeh.2023.114408 37949307

